# Effectiveness analysis of deceleration capacity and traditional heart rate variability in diagnosing vasovagal syncope

**DOI:** 10.3389/fcvm.2024.1333684

**Published:** 2024-09-03

**Authors:** Yongzhe Guo, Tao Lin, Nanyu Lin, Huizhong Lin

**Affiliations:** ^1^ Department of Cardiology, Fujian Heart Medical Center, Fujian Institute of Coronary Heart Disease, Fujian Medical University Union Hospital, Fuzhou, China; ^2^ Department of Epidemiology, School of Public Health, Fujian Medical University, Fuzhou, China

**Keywords:** DC, HRV, SDNN, TTT, VVS

## Abstract

**Background:**

Vasovagal syncope (VVS) is a prevalent medical condition with a lack of efficient methods for its detection.

**Aim:**

This study aimed to explore an objective clinical indicator in diagnosing VVS.

**Methods:**

The retrospective analysis involved clinical data of 243 syncope patients from 1 June 2020 to 31 July 2023. Among them, 108 patients had a negative result in the tilt test (TTT), while the remaining 135 patients had a positive result in the TTT. Relevant statistical methods were utilized to examine the correlation between VVS and different indicators of heart rate variability.

**Results:**

After screening, 354 patients being considered for VVS were evaluated, resulting in a final sample size of 243. Sex, age, deceleration capacity (DC), and standard deviation of all normal-to-normal intervals (SDNNs) were the variables that showed statistical significance between the TTT(−) group and the TTT(+) group. Independent risk factors identified by multivariate logistic regression were DC [odds ratio (OR) 1.710, 95% confidence interval (CI) 1.388–2.106, *P* < 0.001] and SDNN (OR 1.033, 95% CI 1.018–1.049, *P* < 0.001). Comparing the groups, receiver operating characteristic analysis revealed a notable distinction in both DC and SDNN [the respective areas under the curve were 0.789 (95% CI 0.730–0.848) and 0.702 (95% CI 0.637–0.767); the cutoff values were 7.15 and 131.42; *P* < 0.001, respectively].

**Conclusion:**

In summary, DC can function as an impartial and easily accessible clinical marker for differentiating VVS. A value exceeding 7.15 ms might suggest a higher likelihood of syncope.

## 1 Introduction

Vasovagal syncope (VVS) is the predominant reason for fainting in individuals of all age groups (1–4), characterized by an abrupt decline in blood pressure (BP) and/or heart rate (HR). It is distinguished by rapid onset, brief duration, and natural full recovery (5). Due to the high prevalence and lack of efficient medical treatments for VVS (6), numerous patients experience significant physical and psychological distress, resulting in a diminished quality of life (2, 7–10).

The complex pathophysiological mechanisms responsible for vasovagal syncope are still not completely understood. An imbalance in the parasympathetic and sympathetic nerves could have a substantial impact (11–13). Cardioinhibition occurs due to the heightened stress on the parasympathetic nerve of the heart, resulting in bradycardia, asystole, and conduction blockage. Conversely, vasodilation is caused by the inadequate tension of the sympathetic nerve in the blood vessels (14, 15). Several studies have supported the association between VVS and dysfunction of the parasympathetic nervous system (14–21).

In addition to the patient’s medical background and usual clinical symptoms, an objective measure of the performance of the pneumogastric nerve is also a crucial factor in diagnosing VVS. Previous research has yielded inconsistent findings regarding VVS, despite the conventional assessment of heart rate variability (HRV) for analyzing cardiac autonomic function (22, 23). After the initial clinical evaluation, the tilt test (TTT) remains the most valuable diagnostic examination for individuals with suspected reflex syncope (24–26). Vagal modulation has been characterized using the novel measure of heart rate deceleration capacity (DC) (16, 27–30). A decrease in cardiac direct current indicates a decline in the vagal tone of the cardiac autonomic function. Hence, it appears that DC exhibits a higher diagnostic efficacy in individuals with VVS (3, 20, 31). The objective of this research was to determine the involvement of DC and other measures of fundamental autonomic nervous system (ANS) activity in forecasting VVS.

## 2 Materials and methods

### 2.1 Patient recruitment

The institutional research ethics committee of Fujian Medical University Union Hospital approved this retrospective study conducted at a single center. Between June 2020 and May 2023, this hospital gathered clinical information from 354 individuals diagnosed with suspected VVS. VVS was considered if syncope was triggered by fear, pain, or standing up and was accompanied by the usual progressive prodrome (pallor, perspiration, and/or queasiness) (5). The exclusion criteria included (a) heart rhythm abnormalities (paroxysmal supraventricular tachycardia, ventricular tachycardia, atrial fibrillation, Mobitz type Ⅱ second-degree or third-degree atrioventricular block, arrhythmias caused by medication); (b) severe heart or cardiopulmonary conditions (coronary heart disease, cardiac valve diseases, hypertrophic obstructive cardiomyopathy, New York Heart Association class Ⅲ or Ⅳ heart failure, pulmonary hypertension, pulmonary embolism); (c) cerebrovascular disorders (stroke, severe neurological disorders, seizures); (d) syncope caused by medication (antidiabetic drugs, antipsychotics, vasodilators); and (e) conditions affecting the autonomic nervous system (diabetes mellitus, diseases related to the nervous system). The exclusion of patients with orthostatic hypotension was based on the criteria of a minimum decrease of 20 mmHg in systolic blood pressure or 10 mmHg in diastolic blood pressure within the initial 3 min of TTT, as it had been previously demonstrated to impact HRV in a prior study (32). The study included 243 patients who were clinically suspected of having VVS and were screened to rule out any related conditions. These patients underwent echocardiograms, general and biochemical examinations, 24-h Holter recordings, and TTTs after admission.

### 2.2 Tilt test

All TTTs followed the same protocol (33). TTTs were conducted in a softly illuminated chamber following a minimum of 6 h of fasting on an electrically powered table equipped with a footboard. Electrocardiography (ECG) and BP were continuously monitored during the test. The procedure included two stages. Initially, the individuals were inclined at a 70° angle for 30 min (passive stage), followed by an additional 20 min with sublingual administration of 0.25 mg of nitroglycerin (provocative stage) in case the initial stage yielded negative results. TTT was considered positive only if syncope occurred during the testing (5). As per the classification of the Vasovagal Syncope International Study (VASIS) (34), positive findings were categorized into different types. Type 1 (mixed) involved a simultaneous decrease in heart rate and blood pressure, with the heart rate remaining above 40 bpm. Type 2a (cardioinhibition without asystole) indicated a heart rate below 40/min without asystole lasting more than 3 s. Type 2b (cardioinhibition with asystole) referred to a decreased heart rate accompanied by asystole lasting more than 3 s. Type 3 (vasodepressor) was characterized by a rapid drop in blood pressure during syncope, without a decrease in heart rate exceeding 10/min from the baseline. Negative evaluations were assigned to tests with different outcomes.

### 2.3 Holter recording

A minimum of 20 h was spent obtaining 12-channel 24-h Holter data. The digitized recordings were automatically processed using dedicated software (Cardio Care H1200, Nalong Health Technology Co, LTD, Xiamen, China) to determine DC and HRV.

### 2.4 Deceleration capacity

The calculation of DC is based on the phase-rectified signal averaging (PRSA) algorithm, which uses referenced heartbeat intervals (20, 31, 35). Anchors are defined as heartbeat intervals that are longer than the previous interval. To prevent errors caused by artifacts, R-R interval prolongations greater than 5% are excluded. Segments of equal dimensions surrounding the anchors are chosen and aligned with the anchors. Next, the X signals in the aligned segments are averaged. The quantification of DC is determined by the following equation: DC = 1/4 (X_0 _+ X_1 _− X_−1 _− X_−2_), where X_0_ and X_1_ represent the mean values of the anchor points and the subsequent R-R intervals and X_−1_ and X_−2_ denote the averages of the two R-R intervals preceding the anchor points. Daytime DC and nighttime DC were computed for the full 24-h period spanning from 8:00 to 23:00 and from 23:00 to 8:00, respectively.

### 2.5 Heart rate variability

HRV was assessed using five time-domain indexes (36): (a) SDNN, which measures the standard deviation of all normal-to-normal intervals; (b) RMSSD, which calculates the square root of the mean squared differences of successive normal-to-normal intervals; (c) pNN50, which determines the proportion of adjacent R-R intervals differing by more than 50 ms in the 24-h recording; and (d) the frequency domain indices of HRV including the low-frequency (LF) and high-frequency (HF) spectral components, along with the LF/HF ratio.

## 3 Statistical analysis

The data were examined using IBM SPSS version 27.0 in Somers, NY, USA. With this sample size, it was possible to achieve a statistical power of 80% to evaluate a notable distinction (with a margin of error of 0.05). Data normality was assessed using the Kolmogorov–Smirnov test. The normal distribution was followed by all continuous variables, which were expressed as mean ± standard deviation (SD) and examined using an independent-samples *t*-test. The chi-square test and Fisher's exact test were used to compare the case proportion when the expected frequency was less than 5. Multivariate logistic regression analysis was used to investigate the potential link between risk factors and VVS. The discriminatory ability was assessed using the area under the receiver operating characteristic (ROC) curve and its corresponding 95% confidence interval (CI). A two-sided probability value of less than 0.05 was chosen as the threshold for statistical significance.

## 4 Results

### 4.1 Patient characteristics

**Figure 1:**
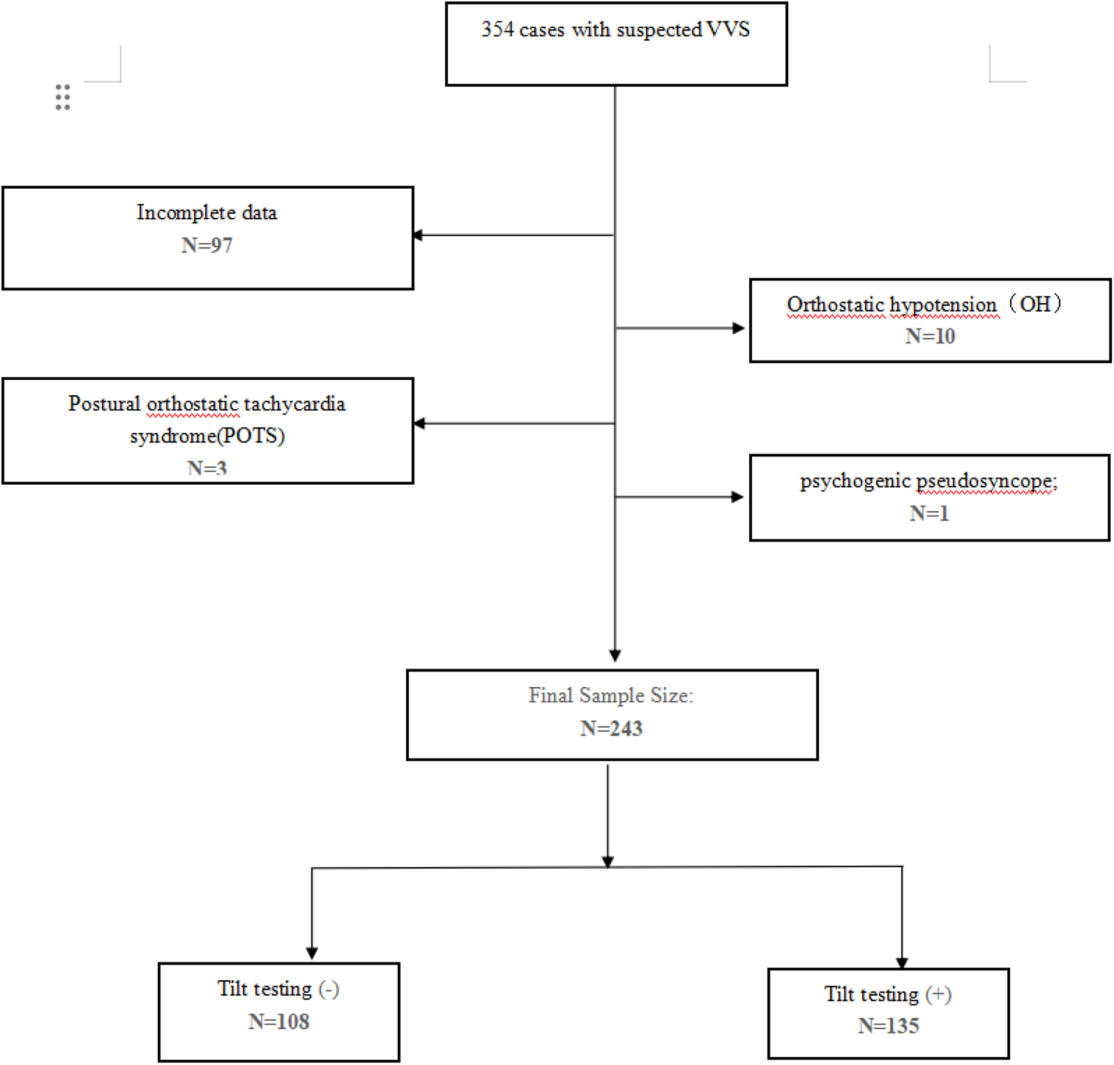
Flowchart of the study patients’ enrollment.

In summary, 354 patients diagnosed with suspected VVS underwent TTT, performed from 1 June 2020 to 31 July 2023 in Fujian Medical University Union Hospital. After removing individuals with orthostatic hypotension (OH), postural orthostatic tachycardia syndrome (POTS), psychogenic pseudosyncope, and incomplete or unavailable data, 243 patients were divided into two groups based on their TTT outcomes (Figure 1). In the TTT(−) group, 108 patients exhibited increased height, reduced weight, lower rates of hypertension and diabetes mellitus, and higher average maximum HR, minimum HR, mean HR, supine SBP, supine DBP, and supine HR. However, no statistical significance was observed in these measurements. In addition, both groups noted a similarity in RMSSD, pNN50, and LF/HF (*P* > 0.05 for all). According to the independent-samples *t*-test, the TTT(−) group reported significantly lower values of DC (6.07 ± 1.76 vs. 8.30 ± 2.33, *P* < 0.05) and SDNN (111.11 ± 22.05 vs. 139.44 ± 46.02, *P* < 0.05) and seemingly higher LF (968.25 ± 1,772.92 vs. 814.79 ± 918.32, *P* = 0.032) and HF (670.82 ± 1,391.01 vs. 419.42 ± 507.0, *P* < 0.05) than the TTT(+) group. Meanwhile, the TTT(−) group showed an older age (53.68 ± 16.67 vs. 48.31 ± 17.33, *P* = 0.015) and a greater proportion of male patients (58.3 vs. 43.7, *p* = 0.023) (Table 1).

**Table 1:** Baseline clinical characteristics of patients stratified by TTT.

	TTT(−) *n* = 108	TTT(+) *n* = 135	*P*-value
Age (years)	53.68 ± 16.67	48.31 ± 17.33	0.015
Male, *n* (%)	63 (58.3)	59 (43.7)	0.023
Height (cm)	162.37 ± 18.05	164.12 ± 8.30	0.318
Weight (kg)	64.06 ± 12.01	61.25 ± 11.38	0.063
Hypertension, *n* (%)	6 (5.6)	10 (7.4)	0.563
Diabetes mellitus, *n* (%)	2 (1.9)	4 (3.0)	0.452
Maximum HR (bpm)	121.73 ± 22.30	120.31 ± 22.13	0.621
Minimum HR (bpm)	49.8 ± 9.27	48.76 ± 7.75	0.345
Mean HR (bpm)	72.78 ± 15.0	70.19 ± 11.38	0.128
Supine SBP (mmHg)	128.01 ± 18.63	124.65 ± 17.38	0.148
Supine DBP (mmHg)	79.55 ± 11.49	77.02 ± 10.88	0.081
Supine HR (mmHg)	70.36 ± 15.65	68.27 ± 12.44	0.248
DC (ms)	6.07 ± 1.76	8.30 ± 2.33	0.000
SDNN (ms)	111.11 ± 22.05	139.44 ± 46.02	0.000
RMSSD (ms)	39.84 ± 39.60	39.80 ± 24.83	0.992
pNN50 (%)	11.09 ± 17.05	11.81 ± 14.64	0.726
LF (ms^2^)	968.25 ± 1,772.92	814.79 ± 918.32	0.032
HF (ms^2^)	670.82 ± 1,391.01	419.42 ± 507.0	0.000
LF/HF	2.31 ± 1.25	2.33 ± 1.19	0.497

DBP, diastolic blood pressure; DC, deceleration capacity; HF, high frequency; HR, heart rate; LF, low frequency; pNN50, percentage of differences exceeding 50 ms between adjacent normal number of intervals; RMSSD, square root of the mean squared differences of successive normal-to-normal intervals; SBP, systolic blood pressure; SDNN, standard deviation of all normal-to-normal intervals.

### 4.2 Logistic regression and ROC curve analyses

**Figure 2:**
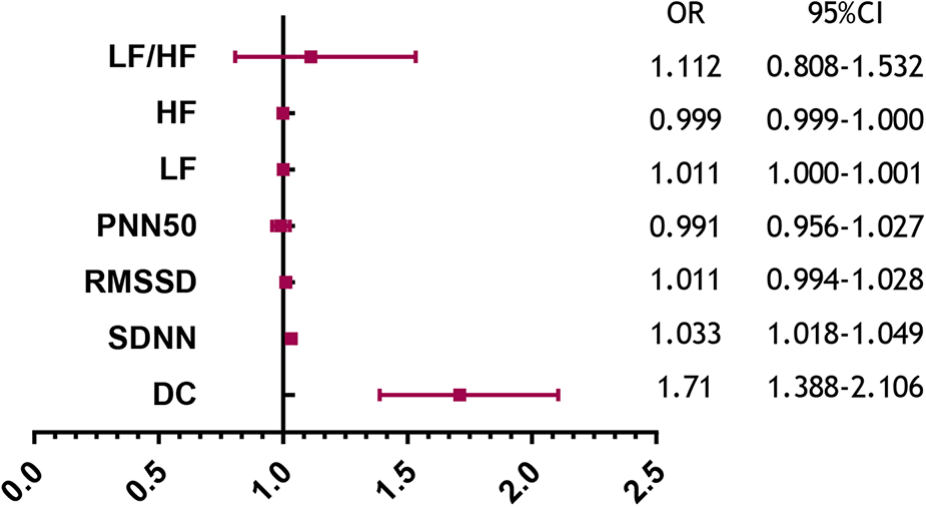
Predictors of the VVS forest plot.

**Figure 3:**
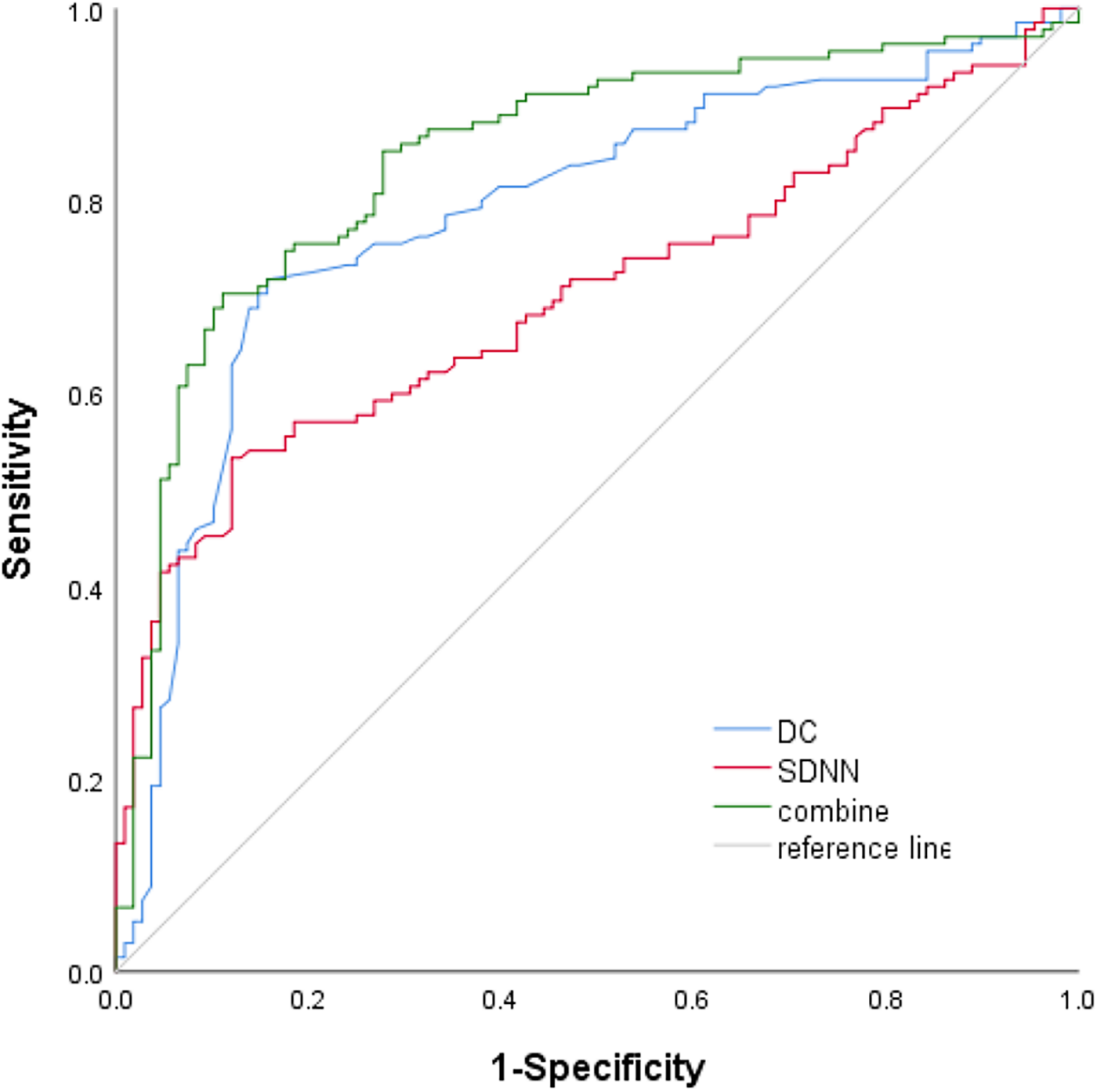
ROC curve for DC, SDNN, and their combination to predict TTT.

Table 2 summarizes the indexes (DC, SDNN, RMSSD, pNN50, LF, HF, and LF/HF) from Table 1 with a significance level of *P* < 0.05. Multivariate logistic regression analysis revealed that VVS was independently associated with DC [odds ratio (OR) 1.710, 95% CI 1.388–2.106, *P* < 0.001] and SDNN (OR 1.033, 95% CI 1.018–1.049, *P *< 0.001). The forest plot (Figure 2) displayed the associations between different variables and VVS, highlighting DC as the factor with the strongest correlation. Figure 3 displays the ROC curve analysis, which helped identify the critical value of continuous variables (DC and SDNN) for detecting patients with VVS. Comprehensive optimization results of sensitivity and specificity were used as the basis for selecting the optimal cutoff point criterion. A clear distinction was noted in DC and SDNN, with corresponding areas under the curve of 0.789 (95% CI 0.730–0.848) and 0.702 (95% CI 0.637–0.767) (*P* < 0.001 for both). According to the ROC curves, the occurrence of VVS was more probable when DC was greater than 7.15 ms or SDNN surpassed 131.42 ms. Table 3 displays all the data related to the ROC curve.

**Table 2:** Multivariable predictors of VVS.

	Multivariate logistic regression analysis		
	OR	95% CI of OR	*P*-value
DC	1.710	1.388–2.106	<0.001
SDNN	1.033	1.018–1.049	<0.001
RMSSD	1.011	0.994–1.028	0.222
PNN50	0.991	0.956–1.027	0.622
LF	1.000	1.000–1.001	0.565
HF	0.999	0.999–1.000	0.262
LF/HF	1.112	0.808–1.532	0.514

DC, deceleration capacity; HF, high frequency; LF, low frequency; pNN50, percentage of differences exceeding 50 ms between the adjacent normal number of intervals; RMSSD, square root of the mean squared differences of successive normal-to-normal intervals; SDNN, standard deviation of all normal-to-normal intervals.

**Table 3:** ROC curve analysis of key indexes between the two groups.

Variables	AUC	95% CI	Critical value	Sensitivity	Specificity	*P*-value
DC	0.789	0.730–0.848	7.15	0.791	0.843	0.000
SDNN	0.702	0.637–0.767	131.42	0.533	0.88	0.000
Combine	0.845	0.795–0.896	—	0.704	0.90	0.000

AUC, area under the curve; DC, deceleration capacity; ROC, receiver operating characteristic; SDNN, standard deviation of all normal-to-normal intervals.

Joint indicators: a combination of DC and SDNN.

## 5 Discussion

Fainting is a prevalent and harmless condition, with a cumulative incidence of at least 35% throughout a person's lifetime and a high likelihood of recurring after the first occurrence (37). Among these fainting cases, approximately 60% are classified as VVS (3, 4), resulting from a combination of various central and peripheral mechanisms (5, 38). As previously stated, VVS is closely linked to dysfunction of both the parasympathetic and sympathetic systems, leading to withdrawal symptoms. To clarify further, triggers that are understood to cause VVS likely lead to vasodilation, resulting in a decrease in the return of blood to the heart and a reduction in the amount of blood in the heart before contraction, as well as a potential decrease in resistance in the outer parts of the body. The low blood pressure linked to VVS could be caused by a lack of narrowing in the small arteries, constriction in the veins, or both (6). Conversely, irregular heart rhythms in VVSs are mainly controlled by the parasympathetic system through the vagal nerve. The heightened activity of the cardiac parasympathetic nerve results in cardioinhibition, resulting in bradycardia, asystole, and conduction blockage (14, 15).

VVS patients believe that heightened parasympathetic activity has a greater role in triggering syncope events (16). HRV refers to the capacity of the heart rate to fluctuate during a specific timeframe and is impacted by the ANS. In recent years, the risk stratification of patients with various diseases has significantly improved due to advancements in parameters related to cardiac autonomic function, measured by HRV (39). A reduced HRV, indicating dysfunction of the cardiac autonomic system, has been linked to an elevated risk of death in various conditions like a heart attack (23, 40, 41), aortic stenosis (42), blood poisoning (43, 44), neurological and psychiatric disorders, blood disorders (45), and even in cancer patients (46, 47). Hence, 24-h Holter monitoring of HRV has emerged as a reliable, non-invasive method for evaluating the ANS, gaining popularity for its practicality and efficiency (48, 49). Nevertheless, prior research has presented contradictory findings regarding VVS (22, 23). Several factors are likely responsible for the inconsistent findings. Analyzing HRV makes it challenging to distinguish the initial impact of vagal and sympathetic modulators on the heart. Furthermore, the absence of a standardized methodology hinders HRV assessments, as the parameters vary depending on factors such as age, gender, physical fitness, sleep quality, and medication use (35, 48, 50).

By analyzing the advantages and disadvantages of HRV, we tried to find some parameters that differed from traditional HRV parameters. Table 1 presents a comparison between DC and SDNN, RMSSD, pNN50, LF, HF, and other commonly used HRV indicators. The findings revealed that the negative group exhibited significantly lower levels of DC and SDNN than the positive group. Moreover, DC displayed a stronger correlation with this disparity than SDNN, with an odds ratio of 1.710 vs. 1.033. This indicated that DC is more effective than conventional HRV in assessing autonomic nervous system function, and this capability seemed to be measurable. Simultaneously, TTT is regarded as a valuable diagnostic instrument for VVS despite its various constraints. The TTT does not meet the desired levels of sensitivity and specificity (51–53). In the present investigation, TTT yielded a positive result in only 55.56% of the individuals who met the clinical diagnostic criteria for VVS. In addition, certain individuals may experience discomfort, particularly during a positive test (3, 20, 31, 54). DC, with its high sensitivity and specificity, addresses the limitations of traditional methods like TTT and HRV while also providing two additional advantages. First, DC allows for the extraction and quantification of the deceleration-induced modulations of the heart rate, enabling a quantitative assessment of vagal tone in patients with VVS. Some studies have shown that DC is consistently modified in patients with VVS (16). Furthermore, the DC algorithm can identify recurring elements of the autonomic regulation process while removing non-recurring elements like disruptive artifacts or irregular heart rhythms. This leads to more consistent and trustworthy assessments in patients with VVS (16). Finally, a recent study indicates that DC seems to have a more robust predictive significance when diagnosing VVS in patients who have a negative TTT response (16).

## 6 Conclusions

The present study found a strong correlation between the cardiac DC index and VVS in patients. A DC value greater than 7.15 ms suggests a considerably higher likelihood of vasovagal syncope.

## Data Availability

The raw data supporting the conclusions of this article will be made available by the authors without undue reservation.
